# Supplementary material for: Biosurfactant-Producing Bacillus velezensis PW192 as an Anti-Fungal Biocontrol Agent against Colletotrichum gloeosporioides and Colletotrichum musae
Source: Microorganisms. 2022 May 12;10(5):1017. doi: 10.3390/microorganisms10051017 (PMC9146131; doi:10.3390/microorganisms10051017)
Supplement: Supplementary file 1 [file microorganisms-10-01017-s001.zip › microorganisms-1718800-supplementary.pdf]

Supplementary

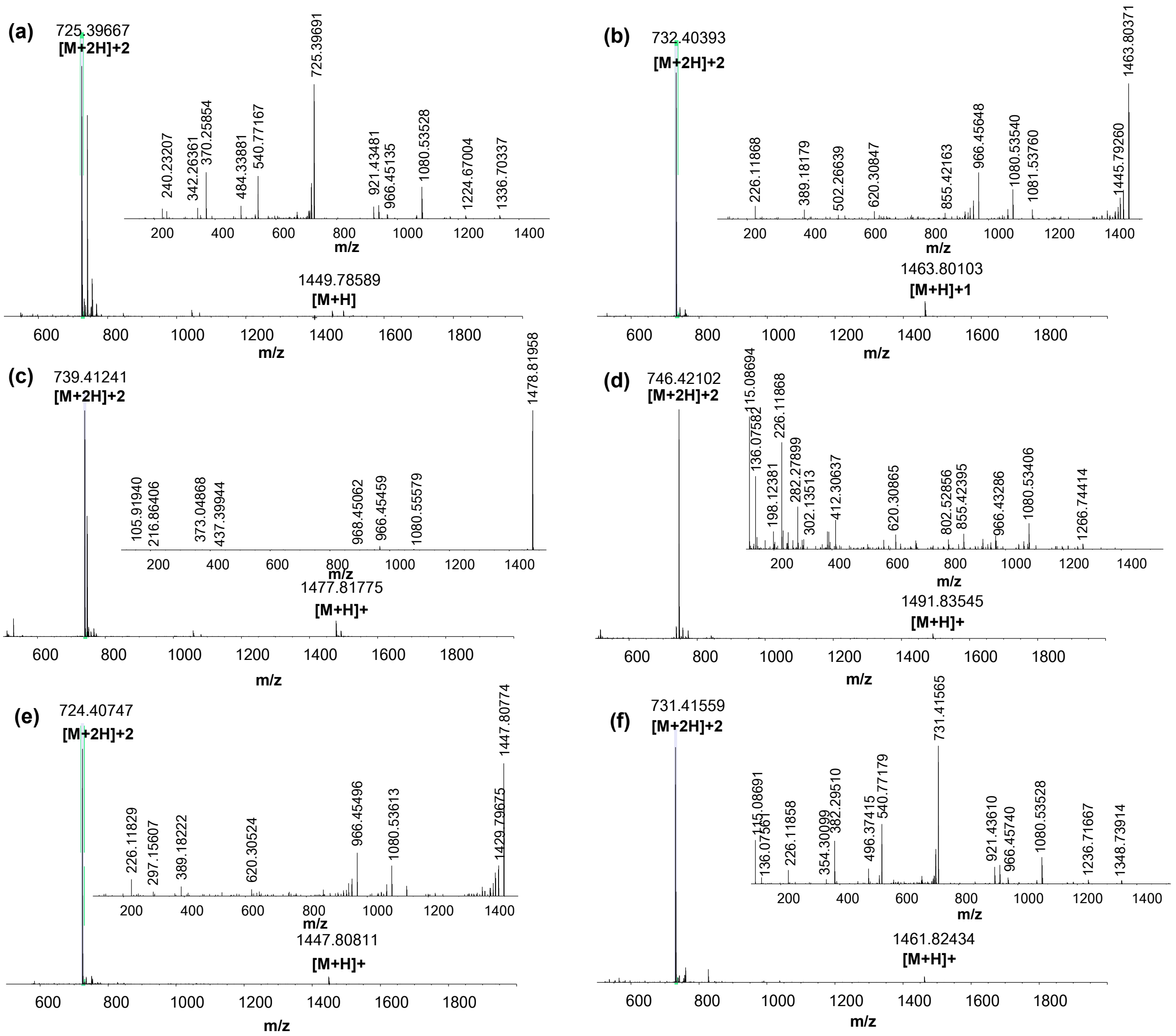

Figure S1. (**a**–**f**) The MS spectra showing doubly and singly charged species of molecular ions of fengycin A derivatives. The insets of (**a**–**f**) illustrate the MS/MS spectra of the molecular ions $[M+2H]^{2+}$ as follows: 725.3967 (inset of (**a**)), 732.4039 (inset of (**b**)), 739.4124 (inset of (**c**)), 746.4210 (inset of (**d**)), 724.4075 (inset of (**e**)) and 731.4156 (inset of (**f**)) using the higher-collision dissociation (HCD) energy of 30 V. The characteristic $[M+H]^{+}$s of 1080.5 and 966.5 is corresponding to Glu-Orn-Tyr-Thr-Glu-Ala-Pro-Gln-Tyr-Ile and Orn-Tyr-Thr-Glu-Ala-Pro-Gln-Tyr-Ile, respectively.

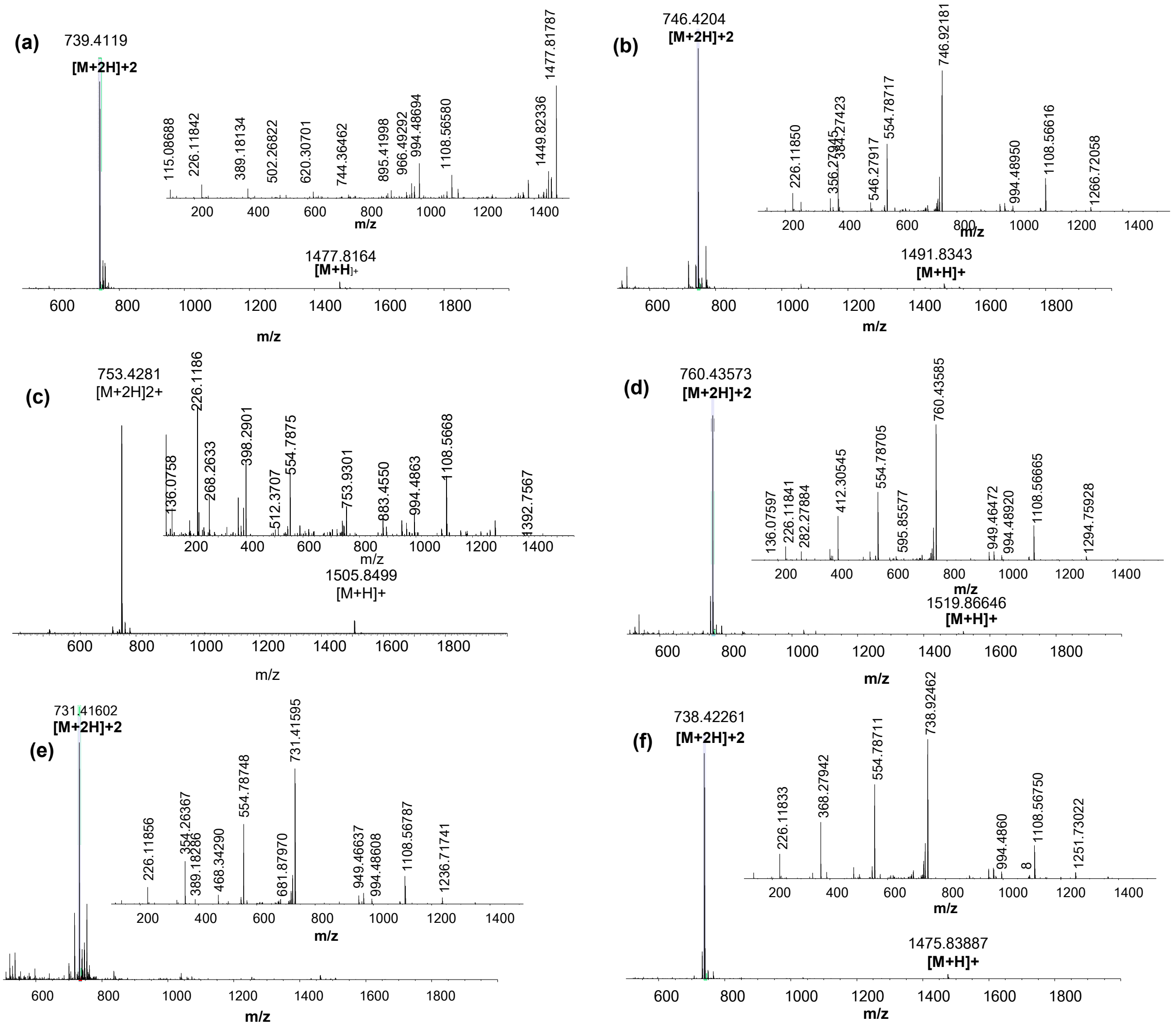

Figure S2. (**a**–**f**) The MS spectra showing doubly and singly charged species of molecular ions of fengycin B derivatives. The insets of (**a**–**f**) illustrate the MS/MS spectra of the molecular ions $[M+2H]^{2+}$ as follows: 739.4119 (inset of (**a**)), 746.4204 (inset of (**b**)), 753.4281 (inset of (**c**)), 760.4357 (inset of (**d**)), 731.4160 (inset of (**e**)) and 738.4226 (inset of (**f**)) using the higher-collision dissociation (HCD) energy of 30 V. The characteristic $[M+H]^{+}$s of 1108.5 and 994.5 is corresponding to Glu-Orn-Tyr-Thr-Glu-Val-Pro-Gln-Tyr-Ile and Orn-Tyr-Thr-Glu-Val-Pro-Gln-Tyr-Ile, respectively.
